# Multiplex CRISPRi System Enables the Study of Stage-Specific Biofilm Genetic Requirements in Enterococcus faecalis

**DOI:** 10.1128/mBio.01101-20

**Published:** 2020-10-20

**Authors:** Irina Afonina, June Ong, Jerome Chua, Timothy Lu, Kimberly A. Kline

**Affiliations:** a Singapore-MIT Alliance for Research and Technology, Antimicrobial Drug Resistance Interdisciplinary Research Group, Singapore; b School of Biological Sciences, Nanyang Technological University, Singapore; c Electrical Engineering and Computer Science, MIT, Cambridge, Massachusetts, USA; d Department of Biological Engineering, MIT, Cambridge, Massachusetts, USA; e Singapore Centre for Environmental Life Science Engineering, Nanyang Technological University, Singapore; Washington University School of Medicine

**Keywords:** Enterococcus faecalis, CRISPR interference, biofilms, gene essentiality, Ebp pili

## Abstract

Enterococcus faecalis is an opportunistic pathogen, which can cause multidrug-resistant life-threatening infections. Gaining a complete understanding of enterococcal pathogenesis is a crucial step in identifying a strategy to effectively treat enterococcal infections. However, bacterial pathogenesis is a complex process often involving a combination of genes and multilevel regulation. Compared to established knockout methodologies, CRISPR interference (CRISPRi) approaches enable the rapid and efficient silencing of genes to interrogate gene products and pathways involved in pathogenesis. As opposed to traditional gene inactivation approaches, CRISPRi can also be quickly repurposed for multiplexing or used to study essential genes. Here, we have developed a novel dual-vector nisin-inducible CRISPRi system in E. faecalis that can efficiently silence via both nontemplate and template strand targeting. Since the nisin-controlled gene expression system is functional in various Gram-positive bacteria, the developed CRISPRi tool can be extended to other genera. This system can be applied to study essential genes, genes involved in antimicrobial resistance, and genes involved in biofilm formation and persistence. The system is robust and can be scaled up for high-throughput screens or combinatorial targeting. This tool substantially enhances our ability to study enterococcal biology and pathogenesis, host-bacterium interactions, and interspecies communication.

## Abstract (precis)

Enterococcus faecalis causes multidrug-resistant life-threatening infections and is often coisolated with other pathogenic bacteria from polymicrobial biofilm-associated infections. Genetic tools to dissect complex interactions in mixed microbial communities are largely limited to transposon mutagenesis and traditional time- and labor-intensive allelic-exchange methods. Built upon streptococcal dCas9, we developed an easily modifiable, inducible CRISPRi system for E. faecalis that can efficiently silence single and multiple genes. This system can silence genes involved in biofilm formation and antibiotic resistance and can be used to interrogate gene essentiality. Uniquely, this tool is optimized to study genes important for biofilm initiation, maturation, and maintenance and can be used to perturb preformed biofilms. This system will be valuable to rapidly and efficiently investigate a wide range of aspects of complex enterococcal biology.

## INTRODUCTION

Enterococci are Gram-positive, opportunistic pathogens that are the second leading cause of hospital-acquired infections (HAIs) (1). Within the *Enterococcus* genus, Enterococcus faecalis and Enterococcus faecium are most commonly isolated from human infection, and E. faecalis is most frequently isolated in HAI (2). E. faecalis causes life-threatening endocarditis, bacteremia, wound infection, and medical device-associated infections, including catheter-associated urinary tract infections (3, 4). Many of these infections are biofilm associated, resulting in their increased tolerance to antibiotic clearance. In addition, enterococci are intrinsically resistant to multiple classes of antibiotics and rapidly acquire resistance through mutation and horizontal gene transfer, further rendering these infections difficult to treat (2, 5). Understanding the mechanisms of biofilm formation, antimicrobial resistance, host immune evasion, and interspecies communication is crucial to more effectively manage and treat enterococcal infections. However, biofilms and antimicrobial resistance involve complex gene pathways comprised of multiple genes, which makes it difficult to study with currently available tools that are designed to study one gene at a time. To date, genetic tools to study enterococcal biology are limited to transposon (Tn) mutagenesis and allelic-exchange gene inactivation or deletion, both of which are laborious, time-consuming, and scalable only in a decelerated step-by-step manner (6–8).

Clustered, regularly interspaced, short palindromic repeat (CRISPR) loci coupled with CRISPR-associated (Cas) proteins were first described to confer bacterial adaptive immunity against bacteriophages and invading plasmids (9–11). Since the repurposing of CRISPR-Cas systems for gene editing, the toolbox for genetic manipulation in bacteria is expanding (12, 13). The well-studied type II CRISPR-Cas system consists of a DNA endonuclease (Cas9) that is guided to the bacterial chromosome by a short 20-nucleotide (nt) single guide RNA (sgRNA), where they generate a double-stranded DNA break by recognizing a 2- to 6-bp DNA sequence called a protospacer-adjacent motif (PAM) that immediately follows the targeted gene sequence (14). The lack of an efficient mechanism for nonhomologous end joining in bacteria makes CRISPR-Cas9 lethal, which inspired the repurposing of CRISPR-Cas for antimicrobial therapy (15–18). CRISPR interference (CRISPRi) takes advantage of a catalytically inactive or “dead” Cas9 (dCas9) that sterically blocks transcription elongation to control gene expression (19, 20). CRISPRi also enables large-scale genome-wide studies and the simultaneous silencing of multiple genes and has been successfully implemented in Escherichia coli, Bacillus subtilis, and Streptococcus pneumoniae, where high-throughput screens identified essential bacterial genes (20–22). The well-characterized dCas9 from Streptococcus pyogenes is generally used for genetic perturbation studies because the Cas9 handle, a 42-nt Cas9-binding hairpin, and the PAM sequence are well defined (13, 19, 23). However, because S. pyogenes dCas9 performance varies in different species, with low knockdown efficiency and proteotoxicity in Mycobacterium tuberculosis, for example, dCas9 from other species such as Streptococcus thermophilus have also been effectively used for CRISPRi (24, 25).

E. faecalis encodes a type II CRISPR-Cas9 system with a canonical PAM of NGG (where N indicates any nucleotide) (26). In E. faecalis, basal levels of chromosomally encoded Cas9 guided to an incoming plasmid via a specific CRISPR RNA and transactivating RNA (cr-RNA–tracrRNA) complex are insufficient to fully prevent the conjugation of a foreign conjugative plasmid (27). However, native chromosomally encoded CRISPR-Cas9 has also been successfully used to target antimicrobial resistance genes *in vivo*, with a significant reduction of the targeted population compared to a Δ*cas9* control (18, 26). The overexpression of Cas9 significantly improves the enterococcal immune capacity by diminishing the rates of plasmid transfer to nondetectable levels *in vitro* (27).

While native chromosomal CRISPR-Cas9 has been used for targeted mutagenesis in E. faecalis, a CRISPRi tool for high-throughput scalable genetic control studies is still lacking. Here, we developed a dual-vector nisin-inducible system for E. faecalis that can efficiently silence single genes and whole operons. This system can also be easily multiplexed to repress multiple genes at the same time. We show that the CRISPRi system can be used to study the genes involved in biofilm formation and antimicrobial resistance as well as essential genes. Importantly, we report effective selective CRISPRi silencing by targeting either the nontemplate or template DNA strand, expanding our understanding of how CRISPRi can work. In addition, we demonstrate the reduction of preformed biofilms through CRISPRi targeting of biofilm-associated genes. The simultaneous silencing of multiple genes and essential genes provides an easily engineered tool to dissect mechanisms of enterococcal pathogenesis, antimicrobial resistance, host-pathogen interactions, and cross-species communication.

## RESULTS

### Construction of a dual-vector CRISPRi system in E. faecalis.

**FIG 1:**
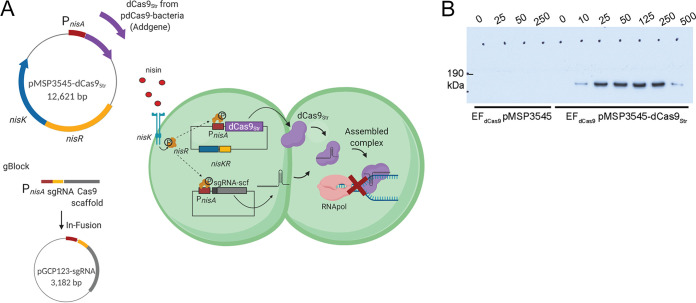
Nisin-inducible dual-vector CRISPRi in Enterococcus faecalis. (A) Schematic diagram of the CRISPRi system in Enterococcus faecalis. Shown is a two-plasmid system consisting of a small (3,182-kb) vector, pGCP123, for sgRNA expression and a 12,621-kb plasmid, pMSP3545-dCas9_Str_, for dCas9_Str_ expression. The sgRNA and dCas9_Str_ are expressed from a nisin-inducible *nisA* promoter that is activated upon the addition of nisin to the medium through the NisKR two-component system encoded on pMSP3545-dCas9_Str_. The assembled sgRNA-dCas9 complex blocks gene transcription by binding to DNA and blocking RNA polymerase. The image was created with BioRender. (B) Western blot with anti-Cas9_Str_ antibody on induced EF_dCas9_ pMSP3545 (empty vector) and EF_dCas9_ pMSP3545-dCas9_Str_ at nisin concentrations of 0 to 500 ng/ml.

To design a scalable CRISPRi expression system, we used the catalytically inactive S. pyogenes Cas9 (dCas9) with its well-defined Cas9 scaffold sequence. We cloned *dcas9* from pdCas9-bacteria (Addgene) under the control of the nisin-inducible promoter *nisA* in pMSP3545, which also encodes the nisin-responsive NisKR two-component system (TCS), to generate pMSP3545-dCas9 (23, 28) (Fig. 1A). Barcoded guide RNA (gRNA) sequences with a dCas9 handle under the control of the same *nisA* promoter were synthesized as gBlocks (IDT, USA) and cloned into the pGCP123 expression vector by an In-Fusion reaction to generate pGCP123-sgRNA (8, 23, 29). Both plasmids were transformed into E. faecalis. Upon the addition of nisin to the medium, NisK is activated and phosphorylates NisR, which binds to the *nisA* promoter to drive the expression of dCas9 from pMSP3545-dCas9 and sgRNA from pGCP123-sgRNA (Fig. 1A) (29). The strength of the *nisA* promoter is dose dependent and peaks at 25 ng/ml of nisin (Fig. 1B).

CRISPR-Cas systems are categorized into six major types (I through VI), with each having a type-specific *cas* gene (30). The CRISPR-Cas system in E. faecalis is a type II system that possesses the type-specific gene *cas9*, which in OG1RF is encoded by *csn1* (OG1RF_10404) (31). Since streptococcal Cas9_Str_ is orthologous to enterococcal Cas9 (NCBI BLASTP) and shares the same PAM, NGG (26, 32), we first tested whether the chromosomally encoded, inactivated E. faecalis Cas9 can recognize the streptococcal dCas9 handle and interfere with the episomal inducible CRISPRi system. To test this, we compared the CRISPRi activity of streptococcal dCas9_Str_ to that of inactivated native enterococcal dCas9_EF_. In E. faecalis SD234 (strain OG1RF expressing *gfp* on the chromosome [33]), we mutated catalytic residues D10A and H852A to generate the strain SD234::dCas9 (EF*_dCas9_*). The two catalytic residues correspond to streptococcal catalytic residues D10 and H840 in the RuvC-I domain and HNH domain, respectively, which are responsible for nontemplate and template DNA strand cleavage (13). We then cotransformed GFP_g2, encoding an sgRNA that targets the chromosomal *gfp* gene, together with either the pMSP3545 empty vector control or pMSP3545-dCas9_Str_, into EF_dCas9_. We monitored the green fluorescent protein (GFP) signal using flow cytometry, and upon nisin induction, we observed that only 1% of cells transformed with p3545-dCas9_Str_ remained GFP positive, compared to 98% GFP-positive cells in the empty vector control (see Fig. S2 in the supplemental material). Since inactivated dCas9 from E. faecalis does not recognize the streptococcal scaffold to silence *gfp* in the empty vector control, nor does it interfere with *gfp* silencing by dCas9_Str_, we reason that the native (catalytically active) enterococcal Cas9 would also not bind to the scaffold since only catalytic and not binding residues are mutated. These results demonstrate that dCas9_EF_ from E. faecalis does not interfere with scaffold recognition, and subsequent gene silencing, by streptococcal dCas9.

### CRISPRi silencing of chromosomal genes via template or nontemplate strand targeting.

**FIG 2:**
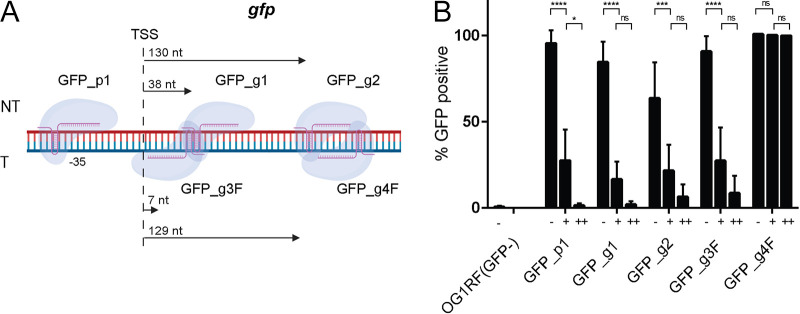
Efficient *gfp* silencing of presensitized cultures on the nontemplate DNA strand. (A) Schematic diagram of the *gfp* operon indicating 5 sgRNAs that target the promoter region (GFP_p1) and protein-coding regions on nontemplate (NT) (GFP_g1 and GFP_g2) and template (T) (GFP_g3F and GFP_g4F) DNA strands. Arrows indicate the distance from the translation start site to the first nucleotide of the bound gRNA. The image was created with BioRender. (B) The 5 sgRNAs were tested for *gfp* repression activity by presensitizing bacteria with nisin overnight and subculturing with nisin induction the next day (++), or bacteria were grown overnight without nisin and induced (+) or not induced (−) the following day. After 2.5 h of subculture, cells were washed and analyzed by flow cytometry. The percentage of GFP-expressing cells was determined by proprietary Attune NxT flow cytometry software from 500,000 events using the EF_dCas9_ p3545-dCas9Str, pGCP123 (EF_dCas9_ pp) empty vector as a 100% positive control. Statistical analysis was performed by 2-way analysis of variance (ANOVA) test using GraphPad. *, *P* < 0.05; ***, *P* < 0.001; ****, *P* < 0.0001; ns, not significant.

We next tested two parameters that can be potentially optimized for efficient CRISPRi targeting, namely, the GC content of the sgRNA and the guide position within the gene and its promoter region (23, 24, 34). We also tested template and nontemplate DNA strand targeting to determine whether only nontemplate strand targeting is efficient in E. faecalis, as has been shown in E. coli, Pseudomonas aeruginosa, Mycobacterium tuberculosis, and Caulobacter crescentus (23–25, 35). We designed guides to test the ability of CRISPRi to silence the *gfp* gene by targeting (i) a promoter region with GFP_p1 (35% GC content), (ii) a nontemplate DNA strand with GFP_g1 (25% GC) and GFP_g2 (20% GC), and (iii) a template DNA strand with GFP_g3F (35% GC) and GFP_g4F (30% GC) (Fig. 2A). To determine the expression conditions for maximal targeting efficiency, we used a saturating concentration of nisin of 50 ng/ml and compared planktonic bacteria subcultured without (−) and with (+) nisin for 2 h. We observed only partial silencing (up to 70%) for 4 of the 5 guides when bacteria were induced for 2 h (Fig. 2B). To improve silencing, we presensitized bacteria with nisin induction overnight before subculturing the bacteria into fresh medium again with nisin for 2 h (++). Presensitizing the bacteria universally increased the silencing efficiency for all active guides from 70% to 99% (Fig. 2B). In contrast to what has been reported for E. coli, P. aeruginosa, and M. tuberculosis, where maximal efficiency was observed upon targeting the nontemplate DNA strand close to the transcription start site, we observed that 4/5 guides, including the template-targeting GFP_g3F, performed similarly under each test condition (Fig. 2B) (23–25). In contrast, sgGFP_g4F, which targeted the template strand at a distance from the translation start site (TSS), exhibited zero silencing and mimicked the empty vector control regardless of the nisin induction time (Fig. 2B). In conclusion, maximal silencing efficiency is achieved with presensitization, where all nontemplate targeted guides perform similarly regardless of the distance from the TSS or the GC content.

### Efficient *ebpABC* operon and selective nontemplate DNA strand silencing in planktonic and biofilm bacteria.

**FIG 3:**
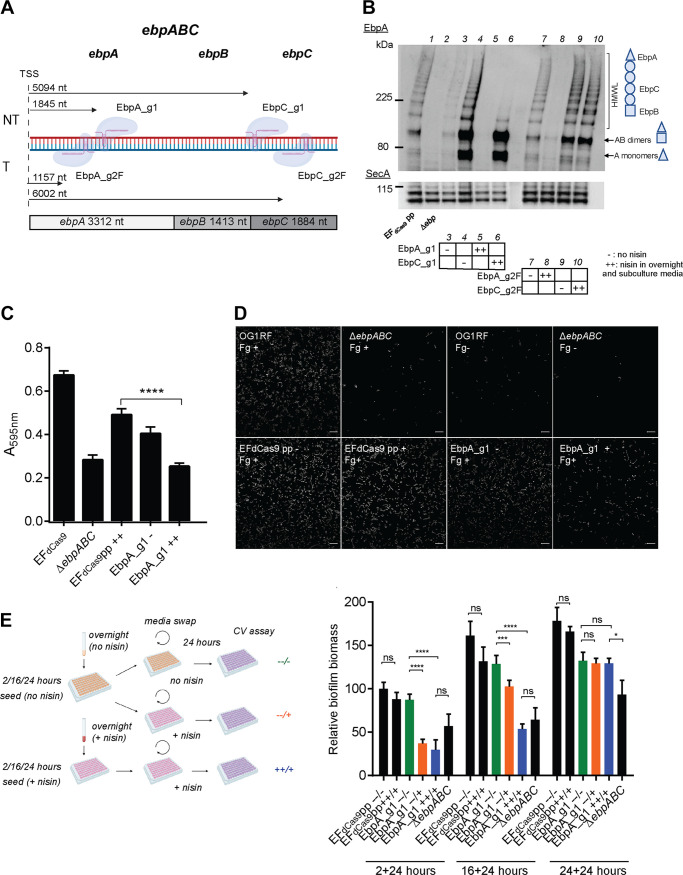

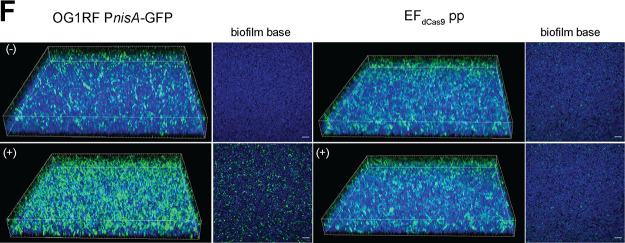
Efficient biofilm perturbation through CRISPRi targeting on *ebpABC.* (A) Schematic diagram of the *ebpABC* operon indicating 4 sgRNAs that target *ebpA* and *ebpC* protein-coding regions on nontemplate (EbpA_g1 and EbpC_g2) and template (EbpA_g2F and EbpC_g2F) DNA strands. Arrows indicate the distance from the translation start site to the first nucleotide of the bound gRNA. (B) Western blot probed with anti-EbpA antibody on whole-cell lysates of *ebpA* and *ebpC* CRISPRi-targeted strains (EbpA_g1 and EbpC_g1 on the nontemplate DNA strand and EbpA_g2F and EbpC_g2F on the template DNA strand) without nisin induction (−) or with nisin induction in cultures grown overnight and subcultures (++). EF_dCas9_ pp with two empty plasmids and Δ*ebpABC* are the control strains. Ebp appears as a high-molecular-weight ladder (HMWL) of covalently polymerized pili of different lengths. SecA served as a loading control and appears as a double band at ∼100 kDa. (C) Crystal violet staining of 24-h biofilms formed on plastic in TSBG medium. EF_dCas9_ pp and Δ*ebpABC* were used as controls, and the test Ebp_g1 strain was uninduced (−) or induced with nisin (50 ng/ml) or presensitized overnight and induced the following day (++) prior to seeding into biofilm chambers. Statistical analysis was performed by the unpaired *t* test using GraphPad. ****, *P* < 0.0001. (D) Confocal images of bacteria attached to fibrinogen-coated biofilm chambers after 2 h of incubation at 37°C. Bacteria were stained with Hoechst DNA dye. EbpA_g1 +, induced; EbpA_g1 −, uninduced; EF_dCas9_ pp, empty vector. The OG1RF and Δ*ebpABC* strains on Fg-coated (Fg^+^) and uncoated (Fg^−^) chambers served as additional controls. Bars, 10 μm. (E) Crystal violet (CV) staining on EbpA_g1 biofilms that were pregrown on plastic without nisin induction for 2, 16, and 24 h, followed by a medium swap with nisin, and left to grow for an additional 24 h (−−/+) (orange bars). Constitutively induced cultures are indicated as ++/+ (blue bars), and constitutively uninduced cultures are indicated as −−/− (green bars), where −−/− indicates no nisin in cultures grown overnight, seed cultures, or swap medium; −−/+ indicates no nisin in cultures grown overnight or in seed cultures but with nisin present in the swap medium; and ++/+ indicates that nisin is present in the medium of cultures grown overnight, upon seeding, and in the swap medium. A swap is indicated by “/.” Statistical analysis was performed by the unpaired *t* test using GraphPad. ****, *P* < 0.0001; ***, *P* < 0.001; *, *P* < 0.05; ns, not significant. (F) Confocal images of biofilms pregrown in plastic chambers for 16 h without nisin induction, followed by a medium swap without (−) or with (+) nisin and subsequent growth for 5 h to allow *gfp* expression and GFP signal maturation. OG1RF P*nisA*-GFP carries *gfp* under the control of P*nisA* from pGCP123, and *nisRK* is carried on the pMSP3545 plasmid. EFdCas9 pp constitutively expressing GFP from the chromosome is the control strain. Bacteria were washed and stained with Hoechst dye prior to taking the Z-stack images. Imaris software 3D modeling of the confocal laser scanning microscopy images is on the left, and the corresponding first (bottom) layer of the Z-stack is on the right. Bars, 10 μm.

To explore the efficiency of CRISPRi silencing of whole operons, we designed sgRNAs to target the *ebpABC* operon, which encodes endocarditis- and biofilm-associated pili (Ebp) important for biofilm formation (36, 37). The *ebpABC* operon is comprised of *ebpA*, *ebpB*, and *ebpC* expressed from the same promoter upstream of *ebpA* (38). EbpC is the major pilin subunit, EbpA is the tip adhesin, and EbpB is found at the base of the polymerized pilus (38). In the absence of EbpA, long pili are still polymerized, in which EbpC comprises the stalk of the pilus, while in the absence of EbpC, only short EbpA-EbpB dimers are formed (39). To test whether silencing the first gene in the operon could effectively silence the entire operon, we designed EbpA_g1 and EbpC_g1, which target the nontemplate protein-coding DNA strand 1,845 and 5,094 nt downstream of the *ebpA* TSS (Fig. 3A). Since we observed selective efficiency of *gfp* template DNA strand targeting, to further explore this selectivity, we also designed EbpA_g2F and EbpC_g2F, which target the template DNA strand 1,157 and 6,002 nt downstream of the *ebpA* TSS, respectively (Fig. 3A). We assessed the efficiency of operon transcriptional silencing by quantifying the amount of polymerized pili by Western blotting and by quantifying pilus function in biofilm formation since pilus-deficient strains are attenuated for biofilm formation (37).

Both guides that target *ebpA*, the first gene of the *ebpABC* operon, regardless of the targeted DNA strand, were similarly efficient in silencing the whole operon as measured by the absence of a high-molecular-weight ladder (HMWL) by Western blotting (Fig. 3B, lanes 5 and 8), indicating a lack of pilus polymerization. As expected, the lack of polymerized pili after EbpA_g1-mediated silencing correlated with reduced biofilm formation, similar to an *ebpABC*-null mutant (Fig. 3C). EbpC_g1 targeting the nontemplate strand efficiently silenced *ebpC* transcription but did not affect the expression of the upstream *ebpA* and *ebpB* genes, resulting in the absence of HMWL Ebp but the presence of EbpAB dimers (Fig. 3B, lane 6). In contrast, EbpC_g2F did not silence *ebpC*, leaving pilus expression unaffected (Fig. 3B, lane 10). Therefore, taken together with the *gfp* targeting data described above, these data suggest that the silencing efficiency achieved by targeting the template DNA strand varies and, at least in these two instances, is less effective when the target is farther away from the TSS. In contrast, targeting the nontemplate strand is efficient to silence a single gene or the whole operon and can act at a significant distance from the operon TSS.

While Ebp is important for biofilm formation, EbpA itself can bind host fibrinogen via the amino-terminal domain of EbpA (40, 41). Since biofilm formation is a complex multifactorial process, we tested if CRISPRi of *ebpA* can also prevent adherence of the bacteria to fibrinogen. We precoated biofilm chambers with fibrinogen, silenced *ebpA* by inducing EbpA_g1, and performed fibrinogen-binding assays as described previously (40). We observed few attached cells in EbpA_g1-induced (EbpA_g1^+^) cells compared to empty vector controls or uninduced (EbpA_g1^−^) bacteria (Fig. 3D, bottom). OG1RF and OG1RF Δ*ebpABC* on fibrinogen-coated (Fg^+^) or uncoated (Fg^−^) chambers served as positive- and negative-control strains, respectively (Fig. 3D, top).

Biofilm formation proceeds via a series of developmental steps, starting with the adhesion of a single cell, aggregate or microcolony formation, maturation into a heterogeneous three-dimensional (3D) structure, and, ultimately, dispersal (42). Single-gene knockouts or *a priori* gene silencing enables the study of the contribution of gene products to biofilm adhesion or initiation; however, the study of genes specific to postadhesion steps of biofilm formation has been more challenging to achieve. Since *ebpABC* are important in biofilm formation, we tested if CRISPRi can be used to perturb preformed biofilms to assess pili involvement in E. faecalis biofilm maintenance. We allowed biofilms to form with uninduced bacteria for 2, 16, or 24 h; subsequently swapped the medium to fresh medium with or without nisin; and incubated the biofilms for another 24 h. We observed a significant decrease in biofilm formation when nisin was added to silence pilus gene expression (−−/+) compared to uninduced biofilms (−−/−) in early (2-h) and 16-h pregrown biofilms (Fig. 3D). Constitutively suppressed Ebp (++/+) formed less biofilm than biofilm with Ebp suppression after the uninduced biofilm was performed (−−/+), showing that Ebp is important for both the initiation and maintenance of E. faecalis biofilms. To determine whether nisin can fully penetrate the biofilm, leading to CRISPRi silencing of genes at the base of the biofilm, we constructed an EF*_dCas9_* P*nisA-*GFP strain that expresses *gfp* under the control of the nisin-inducible promoter, and the *nisRK* TCS is encoded on cotransformed pMSP3545. We added nisin to uninduced 16-h preformed biofilms and allowed biofilms to grow for an additional 5 h to express *gfp* and develop the GFP signal. We observed GFP throughout the biofilm, including the biofilm base (see Fig. 3F), indicating that nisin can penetrate the preformed biofilm. These data demonstrate that inducible CRISPRi can be used to probe stage-specific gene contributions to biofilm maturation and maintenance.

### CRISPRi silencing of *croR* mimics a *croR*::Tn phenotype in antibiotic sensitivity assays.

The CroRS two-component system contributes to E. faecalis antibiotic resistance, survival within macrophages, stress responses, and growth (43–47). CroR phosphorylation by the cognate CroS sensor kinase is important for resistance to bacitracin, vancomycin, and ceftriaxone, where Δ*croR* cells are no longer resistant to these antibiotics (44). To further validate the CRISPRi system in E. faecalis, we designed CroR_g1 to target *croR* on the nontemplate DNA strand and assessed the sensitivity of the resulting strain to bacitracin, using *croR*::Tn as a control. Nisin-induced CroR_g1 bacteria mimicked *croR*::Tn and showed reduced resistance to bacitracin (MIC, 8 μg/ml) compared to the empty vector control (MIC, 32 μg/ml) (Table 1). Hence, the CRISPRi system can be used to study genes involved in antibiotic resistance.

**TABLE 1:** CRISPRi recapitulates the antibiotic sensitivity phenotype of a *croR* mutant^*a*^

Strain	Median bacitracin MIC (μg/ml)
EF_dCas9_ pp (++)	64
*croR*::Tn	4
CroR_g1 (−)	32
CroR_g1 (+)	16
CroR_g1 (++)	4

a Bacitracin resistance of EF_dCas9_ pp, *croR*::Tn, and CroR_g1 uninduced (−), induced (+), or presensitized and induced (++) with nisin (50 ng/ml) after 16 h. Median MICs (micrograms per milliliter) from >3 biological replicates are reported.

### Efficient combinatorial gene silencing.

**FIG 4:**
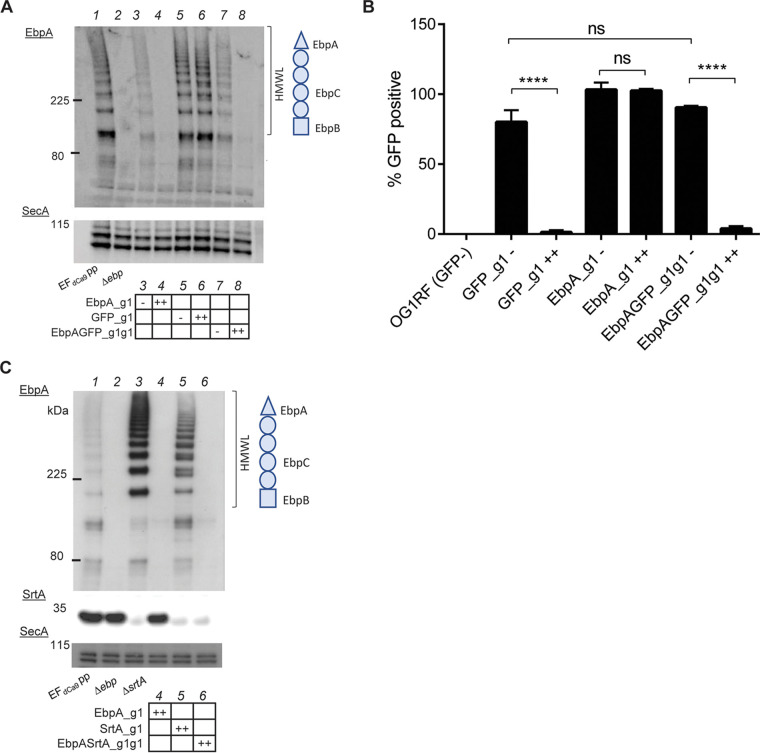
Efficient simultaneous silencing of two different genes. (A) Western blot probed with anti-EbpA antibody on whole-cell lysates of strains expressing EbpA_g1, GFP_g1, or EbpAGFP_g1g1 presensitized and induced cultures. The *ebp*-null and EF_dCas9_ pp strains were used as the control strains. SecA served as a loading control. (B) Percentage of GFP-expressing cells as determined by proprietary Attune NxT flow cytometry software from 500,000 events from 3 independent experiments using the EF_dCas9_ pp empty loading control as a 100% positive control. Statistical analysis was performed by the unpaired *t* test using GraphPad. ****, *P* < 0.0001; ns, not significant. (C) Western blot probed with anti-EbpA and anti-SrtA antibodies on whole-cell lysates of strains expressing EbpA_g1, SrtA_g1, or EbpASrtA_g1g1 presensitized and induced cultures. The EF_dCas9_ pp and Δ*srtA* strains were used as the control strains. SecA served as a loading control.

We next assessed the ability of the CRISPRi system to simultaneously silence multiple genes. At the same time, we tested if the presence of a second guide with the same *nisA* promoter and Cas9 handle could be unstable or prone to recombination and interfere with the efficiency with CRISPRi, as has been reported for some systems (48, 49). We used combinatorial genetic *en masse* (CombiGEM) technology (50) to generate GFPEbpA_g1g1 and EbpASrtA_g1g1, enabling the expression of two sgRNAs under the control of independent *nisA* promoters, to simultaneously silence the gene pair *gfp* and *ebpA* or *ebpA* and *srtA*. SrtA is an enzyme that covalently attaches polymerized pili to the cell wall, and the deletion of this gene results in the absence of Ebp bound in the cell wall fraction of cells (38). Upon nisin induction of GFPEbpA_g1g1, we observed the simultaneous loss of the EbpA signal by Western blotting (Fig. 4A, lane 8) and reduction of the GFP signal by flow cytometry (Fig. 4B). Similarly, in the induced EbpASrtA_g1g1 strain, no signal was observed for EbpA or SrtA by Western blotting (Fig. 4C, lane 6) compared to the empty vector control (Fig. 4C, lane 1). Thus, the combinatorial plasmids silenced two gene pairs with an efficiency equivalent to that of single-guide gene silencing, where the presence of a second sgRNA construct did not affect the silencing efficiency of the other guide.

### Essential gene targeting with presensitizing at subinhibitory nisin concentrations.

**FIG 5:**
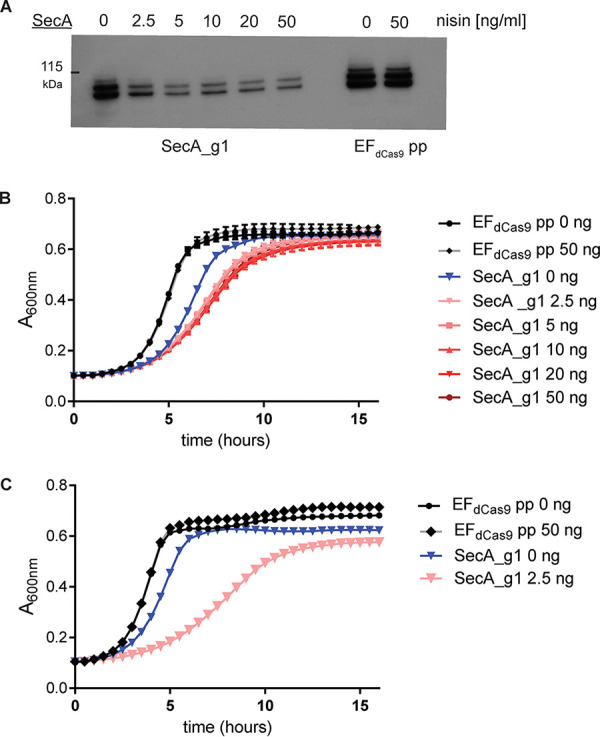
Essential gene targeting with presensitizing at subinhibitory nisin concentrations. (A) Western blot probed with anti-SecA antibody on whole-cell lysates from the strain expressing SecA_g1 grown for 3 h after subculture with nisin at concentrations of 0 to 50 ng/ml. (B) Growth curves of SecA_g1 induced at various nisin concentrations (0 to 50 ng/ml) without overnight presensitization with EF_dCas9_ pp (empty load) as a control. (C) Growth curves of SecA_g1 presensitized overnight with 2.5 ng/ml of nisin prior to subculture in fresh medium with nisin at 2.5 ng/ml. EF_dCas9_ pp (empty load) without induction and with presensitization and induction at 50 ng/ml was the control.

Finally, we assessed the ability of the inducible CRISPRi system to study essential genes. We chose to target *secA*, an essential gene of the general secretion pathway (51). We designed SecA_g1 to target the nontemplate DNA strand of *secA*. Because *secA* is essential, silencing of this gene is predicted to attenuate the growth of the strain. We performed growth curves at various nisin concentrations to assess growth inhibition after *secA* targeting. Even without induction, we observed a reduced growth rate compared to the empty vector control, presumably due to a leaky *nisA* promoter with basal expression of dCas9_Str_ and SecA_g1 (Fig. 5). Upon induction, we observed similar growth inhibition for nisin concentrations of 2.5 to 50 ng/ml, suggesting that maximal inhibition of SecA without presensitization is achieved at 2.5 ng/ml (Fig. 5B). To increase the degree of inhibition, we presensitized bacteria overnight with 2.5 ng/ml nisin, as we observed no growth for cultures grown overnight at nisin concentrations of 5 to 50 ng/ml (data not shown). When presensitized cultures were further subcultured with 2.5 ng/ml of nisin, we observed a pronounced growth defect, which was most apparent between 4 and 10 h after induction, compared to the empty vector (no-guide) control (Fig. 5C). In summary, we showed that the function of essential genes can be studied using a low-level nisin presensitization and induction protocol.

## DISCUSSION

Genetic tools to easily and rapidly study the contribution of single or multiple enterococcal genes in a given biological process are lacking. To address this, we developed a scalable dual-vector nisin-inducible CRISPRi system for E. faecalis. The system is most efficient on presensitized cultures and can be used to study a variety of bacterial phenotypes, including biofilm formation, antimicrobial resistance, and gene essentiality.

Similar to CRISPRi systems developed for other bacterial species, our system is inducible and efficient and can be multiplexed (21, 24). We employed a two-plasmid system: one plasmid encodes dCas9 and a nisin-responsive two-component system, and the other encodes the nisin-inducible sgRNA. The second plasmid, pGCP123, is small and easily modifiable for combinatorial targeting. We can introduce sgRNA into a digested plasmid in the form of a gBlock (or reannealed oligonucleotides) through Gibson assembly or In-Fusion reactions (22, 50, 52). The plasmid can be further modified for the simultaneous targeting of multiple genes by the ligation-digestion reaction of compatible restriction sites using CombiGEM technology (50). Our system uses the streptococcal dCas9 with the handle, a well-characterized tool for CRISPRi (19, 23). Although Cas9_Str_ is orthologous to native enterococcal Cas9 and shares the same PAM, we showed that the streptococcal dCas9 handle is not recognized by native enterococcal dCas9 and can be used in E. faecalis without modifying the endogenous Cas9 (26, 53).

We observed the highest level of dCas9_Str_ protein expression at 25 ng/ml of nisin. However, nisin is more stable at a lower pH and may partially degrade over 24 h in the culture medium (54). To account for degradation, we used a higher nisin concentration of 50 ng/ml to maintain the maximal strength of the *nisA* promoter (28, 54). At 50 ng/ml of nisin, most Gram-positive and Gram-negative bacteria are able to replicate as the MIC is typically >1,000 ng/ml, allowing our system to be used in the context of multispecies interactions (55). Since the nisin-controlled gene expression system is functional in a wide range of Gram-positive bacteria, including *Lactococcus*, *Lactobacillus*, *Leuconostoc*, *Streptococcus*, and *Enterococcus*, our CRISPRi-Cas9 system therefore can be potentially used in these genera (56).

To design sgRNAs, we used the CHOPCHOP database on the E. faecalis OG1RF genome and selected for the guides with a zero off-target score (57). We tested sgRNAs of various GC contents (20 to 60%), targeting template and nontemplate DNA strands, and at various distances from the translation start site (TSS). The GC content played no role in the efficiency of silencing. All nontemplate DNA strand-targeting sgRNAs were efficient, independent of the distance from the TSS, even though in E. coli, the efficiency of silencing was reported to decrease with increasing distances from the transcription start site (19). Surprisingly, we observed that 2 out of the 4 guides designed on the template DNA strand were still efficient in silencing the targeted gene, whereas it has been generally assumed that CRISPRi sgRNA must target the nontemplate DNA strand for efficient silencing in bacteria (19–21, 23). It is possible that template DNA strand-targeting guides, such as GFP_g3F, that bind to the template strand just 7 nt away from the TSS may allow dCas9 to interfere with the assembly of the transcription machinery, preventing transcription initiation. Consistent with this possibility, another guide, GFP_g4F, that targets the template strand 200 nt downstream from the TSS does not silence GFP. However, targeting the template strand of *ebpA* using EbpA_g2F, which binds 1,157 nt away from the TSS, is efficient in gene silencing, indicating that template strand targeting and silencing are not universally distance dependent. Further work is needed to understand the nature and mechanism of template DNA strand silencing in E. faecalis.

A great challenge in studying gene contributions to different stages of developmental cycles, such as those that occur during biofilm formation, arises when early steps are essential for later steps to occur, necessitating the ability for stage-specific gene silencing. We leveraged the inducibility of our system to trigger *ebpA* silencing in preformed biofilms to address the role of these pili after biofilm initiation, during the maturation and maintenance stages. We showed that nisin can penetrate the entire biofilm to trigger gene expression from the *nisA* promoter. We observed a significant reduction in biofilm biomass in the induced cultures compared to uninduced controls, indicating that CRISPRi-Cas9 can be used to perturb the preformed biofilms and to identify and interrogate gene targets in a biofilm stage-specific manner.

To expand the uses of this CRISPRi system, we utilized CombiGEM technology to generate, in one easy step, combinatorial plasmids to target two genes simultaneously (50). We showed that the simultaneous expression of two guides was as efficient in silencing as the expression of a single guide per cell. Despite the presence of the same promoter sequence on different plasmids, we did not observe disruptive recombination of the promoters or between the guides, with stable and consistent repression of both targeted genes. Therefore, this system has the potential to be scaled up for sgRNA library preparation and high-throughput combinatorial studies.

Finally, our system was tested in the study of essential genes, where targeting the essential *secA* gene with minimal nisin induction significantly impaired bacterial growth, while high concentrations of nisin impeded the growth and killed the bacteria. Genetic tools to study essential genes in E. faecalis are limited to transposon mutant library sequencing approaches and essential gene inactivation with in *trans* complementation (6, 58, 59). Therefore, we can employ CRISPRi to study gene essentiality under various nutrient conditions or leverage upon the systems inducibility and probe essentiality of the genes *in vivo*.

In summary, we have developed and validated an efficient CRISPRi system that can be readily used to study single or a combination of genes involved in different biological processes and can be modified for high-throughput screens, including combinatorial analyses, in E. faecalis. This tool will effectively facilitate the study of E. faecalis pathogenesis and allow the rapid identification of novel targets for future interrogation.

## MATERIALS AND METHODS

### Bacterial strains and medium conditions.

The strains and plasmids used in the study are listed in Table 2. E. faecalis strains were grown statically at 37°C in tryptone soy broth (Oxoid, UK) supplemented with 10 mM glucose (TSBG) for biofilm studies, in Mueller-Hinton broth II (MHB-II; Merck, USA) for bacitracin susceptibility tests, and in brain heart infusion (BHI) medium (Merck, USA) or BHI agar (Merck, USA) for the rest of the experiments. E. coli was grown in Luria-Bertani broth Miller (LB; BD, Difco, USA) at 37°C with shaking at 200 rpm. Erythromycin (100 μg/ml) was used to maintain the pMSP3545 plasmid in E. faecalis, and kanamycin (500 μg/ml for E. faecalis and 50 μg/ml for E. coli) was used to maintain pGCP123 and its derivatives. A nisin (Sigma, USA) stock solution was prepared as 0.1 mg/ml by dilution in deionized water. The nisin solution was then filter sterilized through a 0.22-μm filter, aliquoted, and frozen at −20°C. When needed, an aliquot was thawed and used once.

**TABLE 2:** Bacterial strains used in the study

Strain or plasmid	Description	Reference
Bacterial strains		
OG1RF	Human oral isolate OG1	62
SD234	GFP-tagged OG1RF	33
SD234::dCas9	Catalytically inactive dCas9; mutations D10A and H840A	This study
Δ*ebpABC*	Contains an in-frame deletion of the *ebpABC* operon	38
*croR*::Tn	Transposon mutant of *croR*	7
Δ*srtA*	Contains an in-frame deletion of the *srtA* gene	60

Plasmids		
pGCP123	Small shuttle vector for sgRNA expression	8
pGCP213	Temp-sensitive integration vector for allelic exchange	8
pMSP3545	Encodes the nisin two-component system and nisin-inducible promoter *nisA*	28; gift from Gary Dunny (Addgene plasmid 46888)
pMSP3545-dCas9_Str_	Encodes dCas9_Str_ under the control of nisin-inducible promoter *nisA*	This study
pdCas9-bacteria	Catalytically inactive Cas9 from Streptococcus pyogenes	23; gift from Stanley Qi (Addgene plasmid 44249)
pGCP123-P*nisA*-GFP	Contains *gfp* under the control of P*nisA* on the pGCP123 backbone	This study

### Genetic manipulations.

To abolish the endonuclease activity of native enterococcal Cas9_EF_, we first aligned *csn1* (OG1RF_10404) to S. pyogenes Cas9 (BLASTP, NCBI) and identified conserved catalytic D10 and H852 residues within the RuvC-1 active site and HNH endonuclease domains, respectively (13). Both amino acids are essential for cleaving the template and nontemplate DNA strands (13). To create D10A and H852A substitutions and introduce a 1-kb flanking region into Cas9_EF_ for subsequent allelic exchange, we amplified 3 fragments from the bacterial chromosome with primers pairs 1/2, 3/4, and 5/6, listed in Table 3, and performed splice by overlap extension (SOE) PCR of the 3 fragments with primer pair 1/6. The resulting product was introduced into the PstI/KpnI-digested temperature-sensitive vector pGCP213 by an In-Fusion reaction (TaKaRa Bio, Japan). The resulting pGCP213-dCas9_EF_ vector was verified by sequencing and used for allelic exchange to generate SD234::dCas9 as described previously (8). The presence of both mutations D10A and H852A in the *csn1* gene encoding Cas9_EF_ was verified by PCR and sequencing with primer pairs 7/8 and 9/10.

**TABLE 3:** Primers used in the study

Primer	Primer name	Primer sequence (5′–3′)
1	dCas9_PstI_F1	CCCTGGCTGCAGAGACACAATG
2	dCas9_D10A_R1	CCCTATAGCCAGACCAATAACGTAGTCTT
3	dCas9_D10A_F2	TGGTCTGGCTATAGGGACTAATTCTGT
4	dCas9_H852A_R2	GGGATAATAGCATCAATATCATAGTGAGATA
5	dCas9_H852A_F3	TGATATTGATGCTATTATCCCACAAAGT
6	dCas9_KpnI_R3	ACATTGCTTTGGTACCAGTATCATTC
7	D10A_screen_F	GTTGTTTAGAATAGTCCCAAAAGAAC
8	D10A_screen_R	ATTTCGCGTGACTTTTTTTATCC
9	H852A_screen_F	CTTCAAGAAACCGTTGATTTGGAC
10	H852A_screen_R	TTTCTCCCAATAAGCTTTCATATCC
11	dCas9_Str__F	GAGGCACTCACCATGGATAAGAAATACTCAATAGGCTTAGC
12	dCas9_Str__R	GCTCTCTAGAACTAGTTTAGTCACCTCCTAGCTGACTC
13	PnisA_F	TATCGACGGAAGATCAGTCTTATAACTATACTGACAATAGAAACATTAAC
14	PnisA_R	TTACTCATTGAGTGCCTCCTTATAATTTATTTTGTAGTTCCTTCG
15	GFP_F	GCACTCAATGAGTAAAGGAGAAGAACTTTTCACTGG
16	GFP_R	GTCGTTTTACAGATCCTATTTGTATAGTTCATCCATGCCATGTG

To generate pMSP3545-dCas9_Str_, dCas9_Str_ was amplified from pdCas9-bacteria (Addgene plasmid 44249) using the primer pair 11/12, and the purified product was introduced by an In-Fusion reaction into pMSP3545 (Addgene plasmid 46888) digested with SpeI and NcoI.

To generate sgRNA expression vectors, a 307-bp gBlock (IDT, USA) consisting of the *nisA* promoter linked directly to a 20-nt sgRNA linked to the dCas9 scaffold (see Fig. S1 in the supplemental material) was introduced by an In-Fusion reaction into pGCP123 digested with BglII and NotI. The gBlock contains 4 restriction sites (BglII, BamHI, EcoRI, and MfeI) for the generation of a pairwise library through restriction-ligation reactions and an 8-nt unique barcode for high-throughput screens coupled with amplicon sequencing (Fig. S1) (50). sgRNA sequences were selected using the CHOPCHOP database with a zero off-target score and minimal self-complementarity (0 to 1) (Table 4) (57).

**TABLE 4:** sgRNAs used in the study

sgRNA name	sgRNA sequence	DNA target strand	GC content (%)
GFP_p1	GCTTGCAATTATGCTTGAAA	Template	35
GFP_g1	CATCTAATTCAACAAGAATT	Nontemplate	25
GFP_g2	AGTAGTGCAAATAAATTTAA	Nontemplate	20
GFP_g3F	AAAGGAGAAGAACTTTTCAC	Template	35
GFP_g4F	CTTAAATTTATTTGCACTAC	Template	30
EbpA_g1	ACGCCAGGTGCTTTTCCCGA	Nontemplate	40
EbpA_g2F	AGTGAGTCGAGTTCAAACAG	Template	45
EbpC_g1	AGTGACATTCCCATTTGCAT	Nontemplate	40
EbpC_g2F	ATTCCTACGTTAACGCCAGG	Template	50
CroR_g1	ACTTCCATTCCATCCATGAT	Nontemplate	40
SrtA_g1	GAATCGGTACACTTGGTTGA	Nontemplate	45
SecA_g1	ATTCAAGTATACAGGCATTG	Nontemplate	35

10.1128/mBio.01101-20.1 FIG S1 gBlock sequence used to generate the sgRNA-expressing vector. Shown is the 20-nt sgRNA (N) transcribed from the *nisA* promoter (in green), followed by the Cas9_Str_ scaffold sequence and transcription terminator (in red). Each guide was barcoded with a unique 6-nt barcode (B). The four restriction sites (underlined) were used for genetic assembly by CombiGEM. gBlock is flanked by overhang regions (in black) for an In-Fusion reaction in PstI/KpnI-digested pGCP123. Download FIG S1, TIF file, 0.1 MB. Copyright © 2020 Afonina et al. 2020 Afonina et al. This content is distributed under the terms of the Creative Commons Attribution 4.0 International license.

10.1128/mBio.01101-20.2 FIG S2 Native enterococcal Cas9 does not interfere with nisin-inducible streptococcal dCas9. (A) Percentage of GFP-expressing cells from induced EF_dCas9_ Ebp_g2 with either the empty vector pMSP3545 or pMSP3545-dCas9_Str_ determined by proprietary Attune NxT flow cytometry software from 500,000 events from 3 independent experiments using the EF_dCas9_ pp empty vector control as a 100% positive control. Statistical analysis was performed by the unpaired *t* test using GraphPad. ****, *P* < 0.0001. (B) Representative flow cytometry plots showing GFP expression in induced Ebp_g2 with the pMSP3545 empty vector or pMSP3545-dCas9_Str_. Download FIG S2, TIF file, 0.1 MB. Copyright © 2020 Afonina et al. 2020 Afonina et al. This content is distributed under the terms of the Creative Commons Attribution 4.0 International license.

To construct pGCP123-P*nisA*-GFP, we amplified P*nisA* from pMSP3545 using the primer pair 13/14 and amplified *gfp* from the SD213 chromosome using the primer pair 15/16. The two fragments were introduced into BglII-digested pGCP123 by an In-Fusion reaction and validated by sequencing. The pGCP123-P*nisA*-GFP plasmid was then transformed into OG1RF together with pMSP3545 to generate the OG1RF P*nisA*-GFP strain.

### Flow cytometry.

A single colony was inoculated and grown overnight with or without the addition of nisin. The following day, cultures were diluted 1:30 in 1,160 μl of fresh medium in a 2-ml tube and grown for 3 h statically at 37°C. After incubation, the cells were collected by centrifugation, resuspended in 1 ml phosphate-buffered saline (PBS), and analyzed on an Attune NxT flow cytometer. The percentage of GFP-expressing cells was determined by proprietary Attune NxT flow cytometry software from 500,000 events based on polygonal gating on the EF_dCas9_ empty vector control.

### Western blotting.

A single colony was selected from agar plates, inoculated into liquid medium, and grown overnight with or without nisin induction. Cultures grown overnight were then diluted 1:10 in fresh medium in the presence of antibiotics and nisin where appropriate and grown until mid-log phase. Samples were normalized to an optical density at 600 nm (OD_600_) of 0.6 and pelleted by centrifugation, and the pellet was resuspended in 75 μl of 10 mg/ml of lysozyme in lysozyme buffer (10 mM Tris-HCl [pH 8], 50 mM NaCl, 1 mM EDTA, 0.75 M sucrose) and incubated at 37°C for 1 h. After lysozyme treatment, 25 μl of 4× lithium dodecyl sulfate (LDS) buffer (Invitrogen, USA) was added to the samples, and the samples were heated at 95°C for 10 min and stored at −20°C until analysis.

For immunoblot analysis, 10 μl of each sample was loaded onto a 4-to-12% gradient NuPAGE Bis-Tris minigel for SecA or SrtA or a 3-to-8% gradient NuPAGE Bis-Tris minigel for EbpA and run in 1× morpholinepropanesulfonic acid (MOPS) (for 4 to 12% gels) or Tris-acetate (for 3 to 8% gels) SDS running buffer, respectively, in an XCellSureLock minicell for 50 min at 200 V. Proteins from the gel were then transferred to a membrane using the iBlot dry blotting system. The membrane was then blocked with 3% bovine serum albumin (BSA) in phosphate-buffered saline with 0.05% Tween 20 (PBS-T) for 1 h, with shaking at room temperature (RT). SecA, SrtA, and EbpA were detected using custom antibodies raised in rabbit, mouse, and guinea pig, respectively (8, 60). Cas9 was detected using a monoclonal mouse anti-Cas9 antibody (Abcam). Appropriate IgG secondary horseradish peroxidase (HRP)-conjugated antibodies (all Thermo Scientific, Singapore) were used for detection.

### Biofilm assay.

Bacterial cultures grown overnight were washed and normalized to an OD_600_ of 0.7 as described previously (61). A total of 5,000 CFU/well were inoculated in TSBG in a 96-well flat-bottom transparent microtiter plate (Thermo Scientific, Waltham, MA, USA) and incubated at 37°C under static conditions for 24 h. After the removal of planktonic cells, the adherent biofilm biomass was stained using 0.1% (wt/vol) crystal violet (Sigma-Aldrich, St. Louis, MO, USA) at 4°C for 30 min. The microtiter plate was washed twice with PBS followed by crystal violet solubilization with ethanol-acetone (4:1) for 45 min at room temperature. The quantity of adherent biofilm biomass was measured by absorbance at the OD_595_ using a Tecan Infinite 200 Pro spectrophotometer (Tecan Group Ltd., Männedorf, Switzerland).

### Fibrinogen-binding assay.

Bacterial binding to fibrinogen was assayed as described previously (40), with the following modifications. The strains were grown overnight with or without nisin induction, normalized to an OD_600_ of 1, and washed once with PBS. A total of 200 μl of the normalized culture was added to 8-well biofilm chambers (Ibidi, Germany) precoated overnight with 10 mg/ml human fibrinogen (catalog number F3879; Sigma) at 37°C. Cells were incubated for 1 h at 37°C, washed once with PBS, and stained with Hoechst 33342 (Thermo Fisher Scientific, USA) for 30 min at RT. Cells were washed once with PBS, and visualized in Ibidi chambers after excitation with a 405-nm laser using a Zeiss LSM780 confocal microscope (Zeiss, Germany) with a 63× NA1.4 objective.

### Confocal laser scanning microscopy and 3D reconstruction.

Confocal imaging of biofilms was performed as previously described (36), with the following modifications. Cultures grown overnight were normalized to an OD_600_ of 0.7, and 8 μl was inoculated into each well of an 8-well ibiTreat biofilm chamber (Ibidi, Germany) containing 200 μl TSBG medium, with a final concentration of 10^5^ CFU/ml. Biofilms were preformed at 37°C for 16 h with or without nisin, after which the culture medium was swapped, fresh medium with or without nisin was added, and the biofilms were incubated for 5 h at 37°C. Supernatants were removed, and biofilms were washed once with PBS and stained with Hoechst 33342 (Thermo Fisher Scientific, USA) for 30 min at RT. Biofilms were imaged directly from the chambers by exciting with 405-nm and 488-nm lasers using a Zeiss LSM780 confocal microscope (Zeiss, Germany) with a 63× NA1.4 objective. Z-stacks were collected through the entire biofilm thickness, and 3D reconstruction was performed with Imaris software (Bitplane, USA).

### Bacitracin susceptibility determination.

The MIC of bacitracin was determined in liquid MHB-II medium in a 96-well plate. Twofold serial dilutions from 128 μg/ml to 4 μg/ml of bacitracin were prepared in triplicate from a 512-μg/ml bacitracin stock. Cultures of bacteria grown overnight were normalized to an OD_600_ of 0.7, and 8 μl was inoculated into each well containing 200 μl TSBG medium, with a final concentration of 10^5^ CFU/ml. Plates were incubated at 37°C for 16 h. The next day, the MIC was determined by visually assessing turbidity. The lowest concentration of the antibiotic that prevented growth was recorded as the MIC.

### Growth curve assessment.

Cultures grown overnight were washed in PBS and normalized to an OD_600_ of 0.7. Normalized cultures were inoculated into 200 μl BHI medium at a ratio of 1:25. Three biological replicates and four technical replicates were performed for each culture. The 96-well plates were incubated at 37°C for 16 h using the BioTek Synergy 4 plate reader (BioTek, USA). The optical density was taken at 600 nm at 30-min intervals to determine the growth curve of each culture.

### Statistical analysis.

Statistical analyses were performed using GraphPad Prism software (version 6.05 for Windows). All experiments were performed in at least three biological replicates, and the mean value was calculated. All graphs show the standard deviations from independent experiments. Statistical analysis was performed by the unpaired *t* test using GraphPad. *P* values of <0.05 were deemed significant.
