# Supplementary figures and images for: Multiplex CRISPRi System Enables the Study of Stage-Specific Biofilm Genetic Requirements in Enterococcus faecalis
Source: mBio. 2020 Oct 20;11(5):e01101-20. doi: 10.1128/mBio.01101-20 (PMC7587440; doi:10.1128/mBio.01101-20)

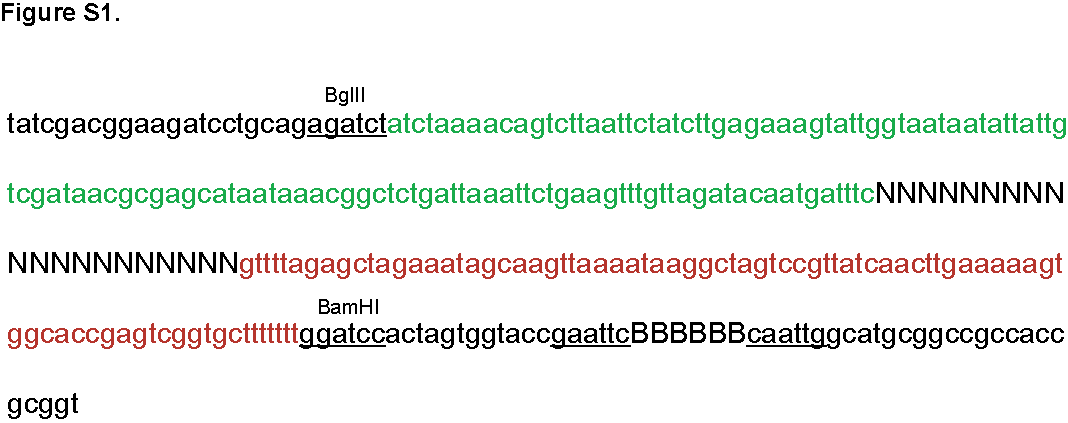

Supplement: FIG S1 [file mBio.01101-20-sf001.tif]

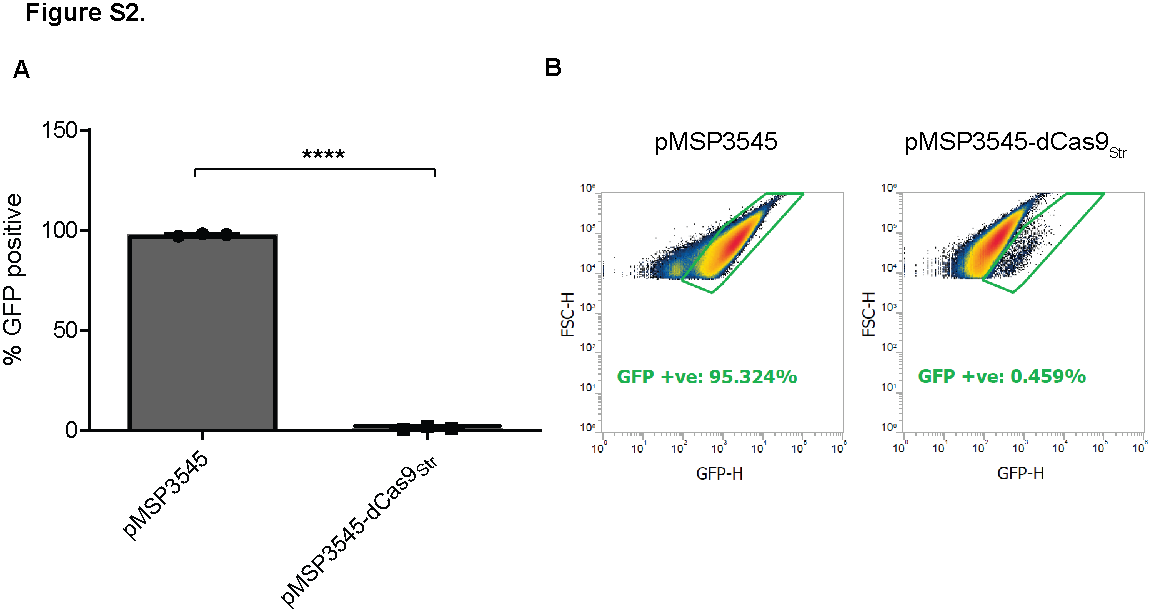

Supplement: FIG S2 [file mBio.01101-20-sf002.tif]
